# Effects of Long-Term Heavy Metal Pollution on Microbial Community Structure in Soil

**DOI:** 10.3390/toxics13090806

**Published:** 2025-09-22

**Authors:** Qiannuo Mi, Yan Wu, Huaisen Cai, Zuben Xu, Yue Zhao, Ronghao Guan, Xin Fan, Jianhua Guo

**Affiliations:** Research Center on Levee Safety and Disaster Prevention of Ministry of Water Resources, Yellow River Laboratory, Yellow River Institute of Hydraulic Research, YRCC, Zhengzhou 450003, China; minn2015@126.com (Q.M.);

**Keywords:** farmland, heavy metal pollution, soil microbial community, heavy metal-tolerant microorganisms, core OTU

## Abstract

Heavy metal (HM) contamination of soil is a worldwide problem with adverse consequences for the environment and human health. Microorganisms, as the most active fraction in soil, play a pivotal role in assessing changes in soil quality and maintaining ecological equilibrium. Accordingly, screening efficient microorganisms for remediating contaminated soils has emerged as a key research focus. This study employed high-throughput sequencing and conducted in situ field surveys to investigate the impacts of long-term HM pollution with varying severity on soil physicochemical properties, as well as the community structure and diversity of bacteria and fungi. The results showed that the major soil physiochemical properties and the bacterial and fungal β diversity significantly changed with the increase in HM pollution levels. The relative abundances of *Chloroflexi*, *Myxococcota* and *Nitrospirota* among bacteria, along with *Chytridiomycota* and *Talaromyces* among fungi, increased significantly with rising HM pollution levels. In low-, medium- and highly contaminated soils, the dominant bacterial species were OTU10618 (*Micrococcales*), OTU6447 (*Chthoniobacterales*), and OTU7447 (*Burkholderiales*), while the dominant fungal species were OTU3669 (*Glomerellales*), OTU397 (*Olpidiales*), and OTU2568 (*Mortierellales*). Bacterial communities were mainly affected by soil-available phosphorus, available cadmium (Cd) and available Pb, while fungal communities were predominantly influenced by soil-available phosphorus, soil organic carbon and total Pb content. These findings demonstrate that soil microorganisms in chronically HM-contaminated soils exhibit adaptive shifts, and this study thereby provides critical implications for assessing the remediation potential of diverse microbial taxa in HM-polluted soils.

## 1. Introduction

The issue of heavy metal (HM) pollution in soil is indeed a critical environmental and public health concern. The primary anthropogenic sources of HM contamination in agricultural soils include industrial wastewater irrigation [1], organic and chemical fertilizers containing excessive HMs, and other industrial emissions [2]. Owing to their high mobility, persistence, and toxicity, HMs can accumulate excessively in agricultural soil and eventually enter the food chain [3,4]. This issue is particularly pronounced in developing countries [5].

Microorganisms are ubiquitous in soil and constitute a major component of the biological community [6,7]. Studies have shown that soil microorganisms are highly sensitive to HM pollution [8]. When HM concentrations exceed a certain threshold, soil quality is altered, primarily manifested through changes in the physiological and ecological properties of soil microorganisms. Elevated HM concentrations significantly impact key microbial community parameters, including abundance, overall metabolic activity, and diversity [9,10]. This suppression of microbial functions likely stems from the additional energy expenditure required to maintain normal growth and metabolic processes under metal stress [11]. However, the way the microbial communities respond to HM exposure is not uniform. Physicochemical properties of soils, such as organic matter content, moisture, pH, and available potassium, play a moderating role in determining microbial responses. Therefore, changes in microbial community structure reflect the combined influence of both HM characteristics (type and concentration) and inherent soil properties [12].

Hunan Province is renowned for its significant abundance of non-ferrous metals, with a total of 140 mineral deposits identified in the region. Among them, W, Sb, Bi, Zn, Pb, and Sn reserves are some of the most plentiful in China, and this region has a rich history of mining. Since July 2014, the People’s Government of Hunan Province has set forth requirements for the centralized treatment of 113,000 hectares of contaminated cultivated land by initiating pilot projects to remediate cultivated land polluted using HMs and adjust crop structures in Changsha, Zhuzhou, and Xiangtan in the Xiangjiang River Basin. This initiative aims to effectively decrease the HM pollution of agricultural products in Hunan Province. In this study, the bacterial and fungal community structures of tailing-contaminated farmland located in Xiangtan (Hunan Province, China) were analyzed comprehensively, in an attempt to provide references and guidance for remedying HM-contaminated farmland.

## 2. Materials and Methods

### 2.1. Sampling Sites

**Figure 1:**
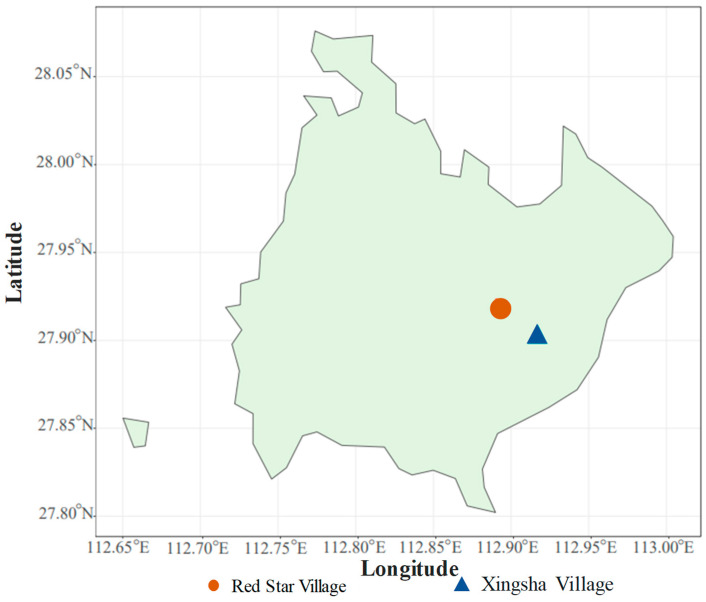
Sampling sites.

Topsoil (0–20 cm depth) was sampled for this investigation. The sampling site was a typical farmland within Yuhu District, Xiangtan, located at a latitude of 27°52′11.9′’ N and a longitude of 112°54′1.03′’ E in Hunan Province, China. The sampling sites can be seen in Figure 1. Characterized by a humid subtropical monsoon climate, the area sees an average annual temperature of 17.2 °C and receives 1320 mm of rainfall. The soil in this region is predominantly laterite with a clay content of 21.7%. Since the early 1980s, the soil in this area has been contaminated by wastewater discharged from nearby Pb-Zn mining operations. Major crops grown in this region during this period include rapeseed, Chinese cabbage, and rice. According to the Soil Environmental Quality Risk Control Standard for Soil Contamination of Agricultural Land (GB15618−2018) [13], when the pH of agricultural soil is between 5.5 and 6.5 (5.5 < pH ≤ 6.5), the risk screening concentration of Cd, Pb, and Zn is 0.3 mg/kg, 90 mg/kg, and 200 mg/kg, respectively; when the pH of agricultural soil is between 6.5 and 7.5 (6.5 < pH ≤ 7.5), the risk screening concentration of Cd, Pb, and Zn is 0.3 mg/kg, 120 mg/kg, and 250 mg/kg, respectively. When the pH of agricultural soil is between 5.5 and 6.5 (5.5 < pH ≤ 6.5), the risk control concentration of Cd and Pb is 2.0 mg/kg and 500 mg/kg, respectively; when the pH of agricultural soil is between 6.5 and 7.5 (6.5 < pH ≤ 7.5), the risk control concentration of Cd and Pb is 3.0 mg/kg and 700 mg/kg, respectively. All these were summarized in Table 1. Given its significant toxicity and concerning pollution status, Cd emerged as the predominant HM pollutant in this study. Consequently, pollution levels were categorized as low, medium, and high based on Cd concentrations. Specifically, low pollution level was indicated by Cd concentrations ranging from 0 mg/kg to 0.03 mg/kg, medium pollution level from 2.39 mg/kg to 4.06 mg/kg, and high pollution level from 4.50 mg/kg to 15.40 mg/kg.

### 2.2. Sample Collection

Soil samples were obtained from Red Star Village and Xingsha Village in Yuhu District, Xiangtan (Hunan Province, China) in April 2019. A total of 22 sampling sites were selected using the “S” type sampling method. Five topsoil samples (0–20 cm) were collected per site with a soil auger. These soil samples were then sieved with a 2 mm mesh and thoroughly mixed to create a composite sample for each site, with at least 2 kg of soil per site. The composite samples were sealed in a ziplock bag and stored in a sampling box. After being transferred to the laboratory, each composite sample was subsampled in triplicate. Among them, 2 subsamples were frozen at −20 °C for the high-throughput sequencing of bacteria and fungi in soil, respectively, while the remaining subsample was air-dried at 25 °C and stored at room temperature for the assessment of soil physicochemical properties.

### 2.3. Determination of Soil Physicochemical Indicators and Heavy Metals

pH: After the soil and water were mixed at a soil-to-water ratio of 2.5:1, the mixture was shaken at 180 r/min for 1 h and then allowed to stand for 30 min, followed by pH determination [14].

Electrical Conductivity: After 5 g of the soil sample was weighed and placed into a plastic centrifuge tube, distilled water was added at a soil-to-water ratio of 1:5. The resulting mixture underwent thorough homogenization through vigorous stirring using a borosilicate glass rod, followed by horizontal oscillation at 200 r/min for 30 min. After allowing the suspension to settle undisturbed for 60 min, the electrical conductivity of the supernatant was measured via a conductivity meter (DDS-11A) [14].

Total Phosphorus: Determined after H_2_SO_4_-HClO_4_ digestion by the molybdenum-antimony colorimetric method [15].

Available Phosphorus: The available phosphorus in soil was extracted using 0.5 mol/L NaHCO_3_ solution and determined by the molybdenum-antimony colorimetric method [16].

Total Nitrogen: Quantified by the Dumas combustion method (Elemental Analyser EA1108, Carlo Erba, Turin, Italy) [17].

Soil Organic Carbon: Measured via the dichromate oxidation and titration method [18].

Available Potassium: Extracted with 1 mol/L NH_4_OAC and quantified by flame photometer (Cole-Palmer 2655-00, Vernon Hills, IL, USA) [19].

Available Cd, Available Pb, and Available Zn: Firstly, a 0.005 mol/L diethylenetriaminepentaacetic acid (DTPA) extraction solution was prepared, containing 0.01 mol/L calcium chloride and 0.1 mol/L triethanolamine, with pH adjusted to 7.30 using hydrochloric acid or ammonia. Exactly 3.0 g of dried soil was mixed with 20 mL of the DTPA solution, followed by shaking, centrifugation, and filtration through a 0.45 μm membrane. Finally, the filtrates were analyzed using ICP–MS (ICP-MS 7700, Agilent Technologies, Santa Clara, CA, USA) within 48 h [14].

Total Cd, Total Pb, and Total Zn: After microwave digestion with a 3:1 HNO_3_/HClO_4_ (*v*/*v*) solution (Start 1500, MLS GmbH, Leutkirch im Allgäu, Germany), the filtrates were determined via inductively coupled plasma mass spectrometry (ICP-MS) (ICP-MS 7700, Agilent Technologies, Santa Clara, CA, USA) [14].

Blanks and a certified reference material, Standard Soil GSS-5, were also prepared by the digestion process for quality assurance. The recovery of the reference material was between 87% and 110%.

### 2.4. High-Throughput Sequencing of Bacteria and Fungi in Soil

Genomic DNA was isolated from 0.5 g of fresh soil samples using the FastDNA Spin Kit for Soil (MP Biomedical, Irvine, CA, USA). DNA concentration and purity were assessed through 1% agarose gel. The 16S rRNA gene in the V3-V4 region and the ITS gene in the ITS1 region were amplified with primers 515F-806R and ITS1F-ITS2R, respectively. The purified amplicons were then sequenced on the Illumina MiSeq sequencing platform (Shanghai Majorbio Bio-Pharm Technology Co., Ltd., Shanghai, China). Operational taxonomic units (OTUs) were defined by clustering sequences at a 97% identity cutoff. The resulting representative sequences for each OTU were then chosen for further taxonomic annotation.

### 2.5. Statistical Analysis

All results are presented as mean ± standard deviation (SD). The diagrams in this study were plotted using Origin8.5. All statistical analyses were performed with IBM SPSS (v20) (IBM Corporation, New York, NY, USA). One-way analysis of variance (ANOVA) was used to compare soil physicochemical properties, bacterial and fungal α-diversity (Chao1 index and Shannon index), and the relative abundance of bacteria and fungi across different HM pollution gradients. Post hoc comparisons were performed using Duncan’s multiple range test (DMRT) with a significance threshold set at *p* < 0.05 to identify specific group differences. To elucidate the relationships between soil physicochemical characteristics and microbial community structures, Canonical correlation analysis (CCA) was performed with the aid of CANOCO (v5.0). The statistical significance of the CCA results was examined through permutation test analysis similar to ANOVA.

The microbiological data were analyzed using the “Vegan” package in R (v4.0.3). Principal coordinates analysis (PCoA) based on the Bray–Curtis distance was performed using the “Vegan” package to evaluate the differences in the composition of bacterial and fungal communities among samples. Permutational multivariate analysis of variance (PERMANOVA), implemented via the “adonis” function, evaluated the effects of HM pollution levels on the composition of bacterial and fungal communities in soil. Bacterial and fungal species with significant differences among microorganisms from soils with different HM pollution levels were identified by linear discriminant analysis Effect Size (Lefse) and analyzed via the diversity cloud analysis platform of Shanghai Majorbio Bio-Pharm Technology Co., Ltd. (https://www.majorbio.com) (accessed 3 September 2022) following a two-stage analytical approach: (1) initial detection of significant taxa through Kruskal–Wallis rank sum tests, followed by (2) effect size estimation with linear discriminant analysis (LDA score > 4.0) to determine biologically meaningful differences [20].

## 3. Results

### 3.1. Effects of Heavy Metal Pollution Levels on Soil Physicochemical Properties

Table 2 demonstrates that with increasing HM pollution severity: (1) electrical conductivity, total phosphorus, available phosphorus, and available potassium showed significant decreases; (2) total Cd, total Pb, available Cd, available Zn, and available Pb concentrations exhibited significant increases; and (3) total nitrogen and organic matter concentrations displayed an initial decrease followed by subsequent increase.

### 3.2. Effects of Heavy Metal Pollution Levels on the α Diversity of Bacteria and Fungi in Soil

Analysis of microbial α-diversity across HM pollution gradients revealed distinct patterns between bacterial and fungal communities.

**Figure 2:**
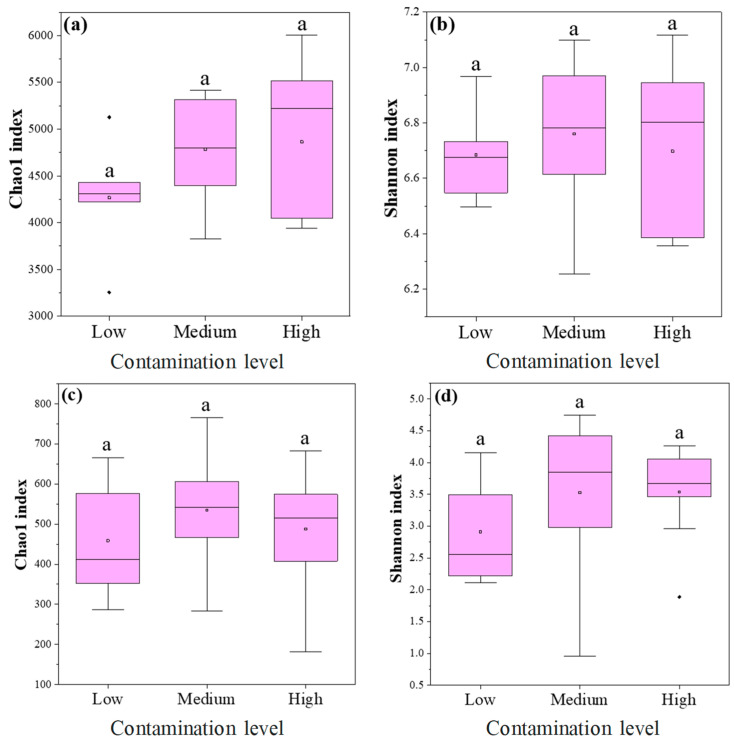
The α diversity of bacterial and fungal communities (**a**,**b**) bacteria; (**c**,**d**) fungi; (**a**,**c**) Chao1 index; (**b**,**d**) Shannon index. Note: Lower-case letters represent the significance of differences across different heavy metal pollution levels (*p* < 0.05).

For bacterial communities, neither the Chao1 index (richness estimator; Figure 2a) nor the Shannon index (diversity metric; Figure 2b) showed statistically significant variation among pollution levels. The highest Shannon index of bacteria was observed in the soil at the medium pollution level, but the difference from that at the low and high pollution levels was not significant (Figure 2b). These findings suggest that bacterial community richness and diversity remained relatively stable despite increasing HM contamination.

For fungal communities, neither the Chao1 index (richness estimator; Figure 2c) nor the Shannon index (diversity metric; Figure 2d) showed statistically significant variation among pollution levels. The highest Shannon index of fungi was observed in the soil at the medium pollution level, but the difference from that at the low and high pollution levels was not significant (Figure 2d). This consistency across pollution gradients indicates that fungal community richness and diversity remained relatively stable despite increasing HM contamination.

### 3.3. Effects of Heavy Metal Pollution Levels on the β-Diversity of Bacteria and Fungi in Soil

**Figure 3:**
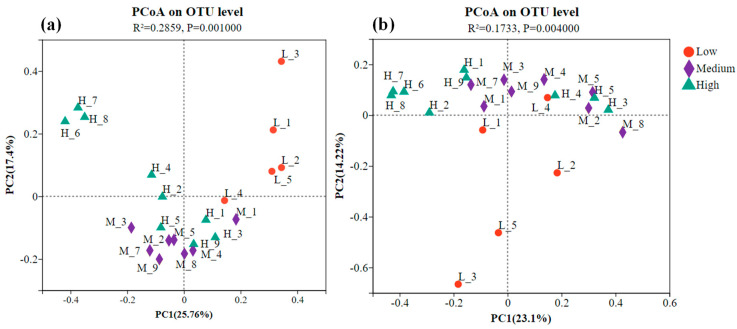
Principal coordinate analysis (PCoA) of the community structure of bacteria (**a**) and fungi (**b**) using the Bray–Curtis distance.

Principal coordinate analysis (PCoA) based on Bray–Curtis distance was performed to examine the similarity or dissimilarity in the structure of bacterial and fungal communities across various HM pollution levels (Figure 3a). The PCoA diagram shows that the first and second principal coordinates explained 25.8% and 17.4% of the total variation, respectively, together accounting for 43.2%. The points in the diagram represent individual samples, with different colors and shapes indicating their respective group affiliations.

As shown in Figure 3, samples from the same pollution level tended to cluster together, indicating similarities in bacterial and fungal community structures under identical pollution conditions. However, substantial heterogeneity among sampling sites suggests that community structure at a given pollution level was influenced not only by HM concentration but also by the specific sampling location.

Adonis (also known as PERMANOVA) was conducted to assess the differences in bacterial and fungal communities across different HM pollution levels (Table 3 and Table 4). The results showed that bacterial communities differed significantly between low and high pollution levels, low and medium pollution levels, and medium and high pollution levels (*p* < 0.05), indicating that HM pollution levels significantly influenced bacterial community structure, in line with the PCoA results. In contrast, fungal communities differed significantly between low and high pollution levels and between low and medium pollution levels, but not between medium and high pollution levels, which is also consistent with the PCoA results.

### 3.4. Effects of Heavy Metal Pollution Levels on the Composition of Bacterial and Fungal Communities

#### 3.4.1. Relative Abundance of Bacterial and Fungal Communities Under Different Heavy Metal Pollution Levels

**Figure 4:**
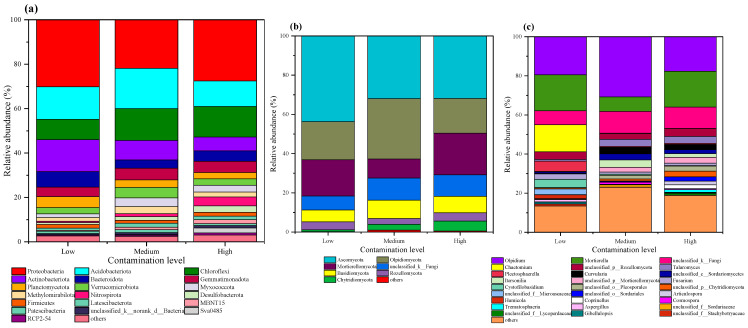
Bacterial community structure at the phylum level (**a**), and fungal community structure at the phylum level (**b**) and the genus level (**c**).

In this study, the rRNA sequences of 22 soil samples under low, medium, and high pollution levels were taxonomically classified using the SILVA database. The bacterial community composition at the phylum level is shown in Figure 4a. Among the bacterial communities, the top 18 phyla each had a relative abundance greater than 1%, while the remaining phyla, with relative abundances below 1%, were categorized as “others”. As shown in Figure 4a, these 18 bacterial phyla included *Proteobacteria, Acidobacteriota*, *Chloroflexi*, *Actinobacteriota*, *Bacteroidota*, *Gemmatimonadota*, *Planctomycetes*, *Verrucomicrobia*, *Myxococcota*, *Methylomirabilota*, *Nitrospirota*, *Desulfobacterota*, *Firmicutes*, *Latescibacteria*, *MBNT15*, *Patescibacteria*, *Sva0485*, and *RCP2-54*. The most dominant phylum was *Proteobacteria*, followed by *Acidobacteriota* and *Chloroflexi*. At low, medium, and high pollution levels, the relative abundances of *Proteobacteria* were 30.2%, 21.9%, and 27.5%, respectively; *Acidobacteriota* accounted for 14.7%, 18.1%, and 11.5%; *Chloroflexi* constituted 9.12%, 14.3%, and 13.7%; *Actinobacteriota* represented 14.4%, 8.77%, and 6.24%; *Bacteroidota* comprised 6.97%, 37.9%, and 4.75%; *Gemmatimonadota* accounted for 4.31%, 5.23%, and 4.99%; Planctomycetes made up 4.93%, 3.42%, and 2.85%; *Verrucomicrobia* constituted 2.82%, 4.71%, and 2.92%; and *Myxococcota* represented 1.68%, 3.82%, and 3.06%, respectively.

The fungal community composition at the phylum and genus levels is shown in Figure 4b and Figure 4c, respectively. At the phylum level, the top seven fungal phyla each exhibited a relative abundance exceeding 1%, while the remaining phyla, each with a relative abundance below 1%, were grouped as “others”. These dominant phyla included *Ascomycota*, *Olpidiomycota*, *Mortierellomycota*, *unclassified_K_Fungi*, *Basidiomycota*, *Chytridiomycota*, and *Rozellomycota*. Under low, medium, and high pollution levels, the relative abundance of *Ascomycota* was 43.7%, 31.9%, and 31.9%, respectively; that of *Olpidiomycota* was 19.5%, 30.8%, and 17.7%; that of *Mortierellomycota* was 18.5%, 9.79%, and 21.3%; that of Basidiomycota was 6.07%, 9.32%, and 8.41%; and that of *Rozellomycota* was 4.03%, 3.04%, and 4.19%. The relative abundance of *Chytridiomycota* increased across pollution levels, measuring 0.99%, 2.98%, and 5.12% in low, medium, and high pollution soils, respectively.

As shown in Figure 4c, the top 16 fungal genera each had a relative abundance greater than 1%, while the remaining genera, each with a relative abundance below 1%, were classified as “others”. These dominant genera included *Olpidium*, *Mortierella*, *Chaetomium*, *Talaromyces*, *Plectosphaerella*, *Curvularia*, *Itersonilia*, *Fusarium*, *Cystofilobasidium*, *Articulospora*, *Humicola*, *Coprinellus*, *Cosmospora*, *Trematosphaeria*, *Aspergillus*, and *Gibellulopsis*. In soils under low, medium, and high pollution levels, the relative abundance of *Olpidium* was 19.5%, 30.8%, and 17.7%, respectively; that of *Mortierella* was 18.3%, 7.35%, and 18.3%; that of *Chaetomium* was 13.9%, 0.01%, and 0.01%; that of *Talaromyces* was 0.83%, 3.57%, and 3.51%; that of *Plectosphaerella* was 5.31%, 0.49%, and 0.53%; and that of *Curvularia* was 0.26%, 3.32%, and 2.69%, respectively.

#### 3.4.2. Relative Abundance of Major Bacterial and Fungal Communities Under Different Heavy Metal Pollution Levels

**Figure 5:**
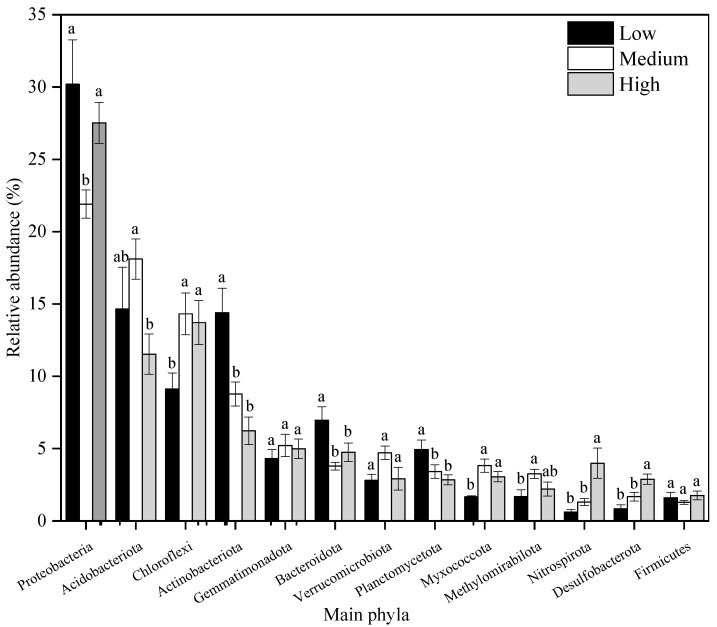
Relative abundance of main bacterial phyla. Note: Lower-case letters represent the significance of differences across different heavy metal pollution levels (*p* < 0.05).

The bar graph of the top 13 bacterial phyla ranked by abundance is shown in Figure 5. Considerable differences were observed in the relative abundances of these phyla across different HM pollution levels. As pollution levels increased, the abundance of *Proteobacteria* decreased initially and then increased significantly, while that of *Acidobacteriota* increased first and subsequently decreased markedly. The abundances of *Chloroflexi*, *Myxococcota*, *Nitrospirota*, and *Desulfobacterota* all increased significantly. In contrast, the abundances of *Actinobacteriota*, *Bacteroidota*, and *Planctomycetes* decreased consistently. No significant differences were observed in the abundances of *Gemmatimonadota*, *Verrucomicrobia*, and *Firmicutes* with increasing pollution levels. Meanwhile, the abundance of *Methylomirabilota* exhibited a fluctuating trend, increasing initially and then decreasing as HM pollution levels rose.

**Figure 6:**
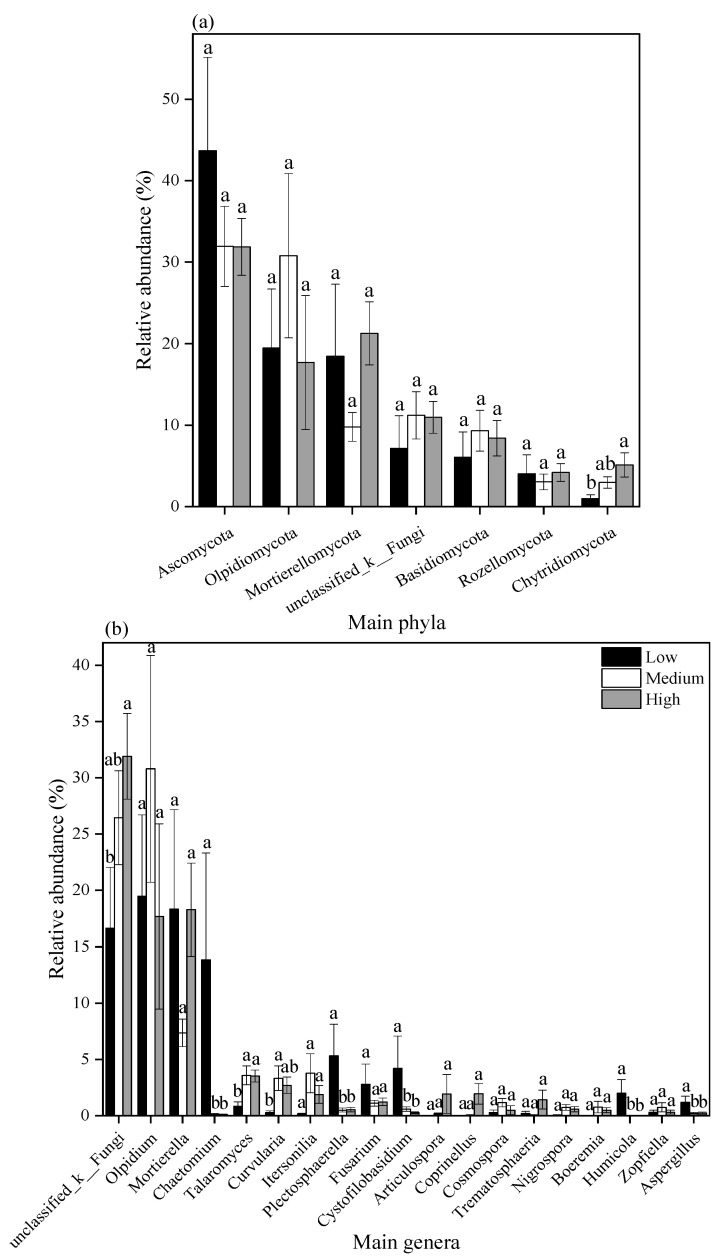
Relative abundance of main fungal phyla (**a**) and fungal genera (**b**). Note: Lower-case letters represent the significance of differences across different heavy metal pollution levels (*p* < 0.05).

Figure 6 shows the relative abundances of the top 7 fungal phyla and the top 19 fungal genera. As seen in Figure 6a, the relative abundance of *Chytridiomycota* increased significantly with increasing HM pollution levels (*p* < 0.05), while no significant changes were observed in the other fungal phyla. According to Figure 6b, the relative abundances of *Chaetomium*, *Plectosphaerella*, *Cystofilobasidium*, *Humicola*, and *Aspergillus* decreased significantly with increasing pollution levels, whereas that of *Talaromyces* increased significantly. No significant changes were detected in the abundances of *Olpidium*, *Mortierella*, *Itersonilia*, *Fusarium*, *Articulospora*, *Coprinellus*, *Cosmospora*, *Trematosphaeria*, and *Gibellulopsis*. The abundance of *Curvularia* exhibited an initial increase followed by a decrease as HM pollution levels rose.

### 3.5. Linear Discriminant Analysis Effect Size (Lefse)

**Figure 7:**
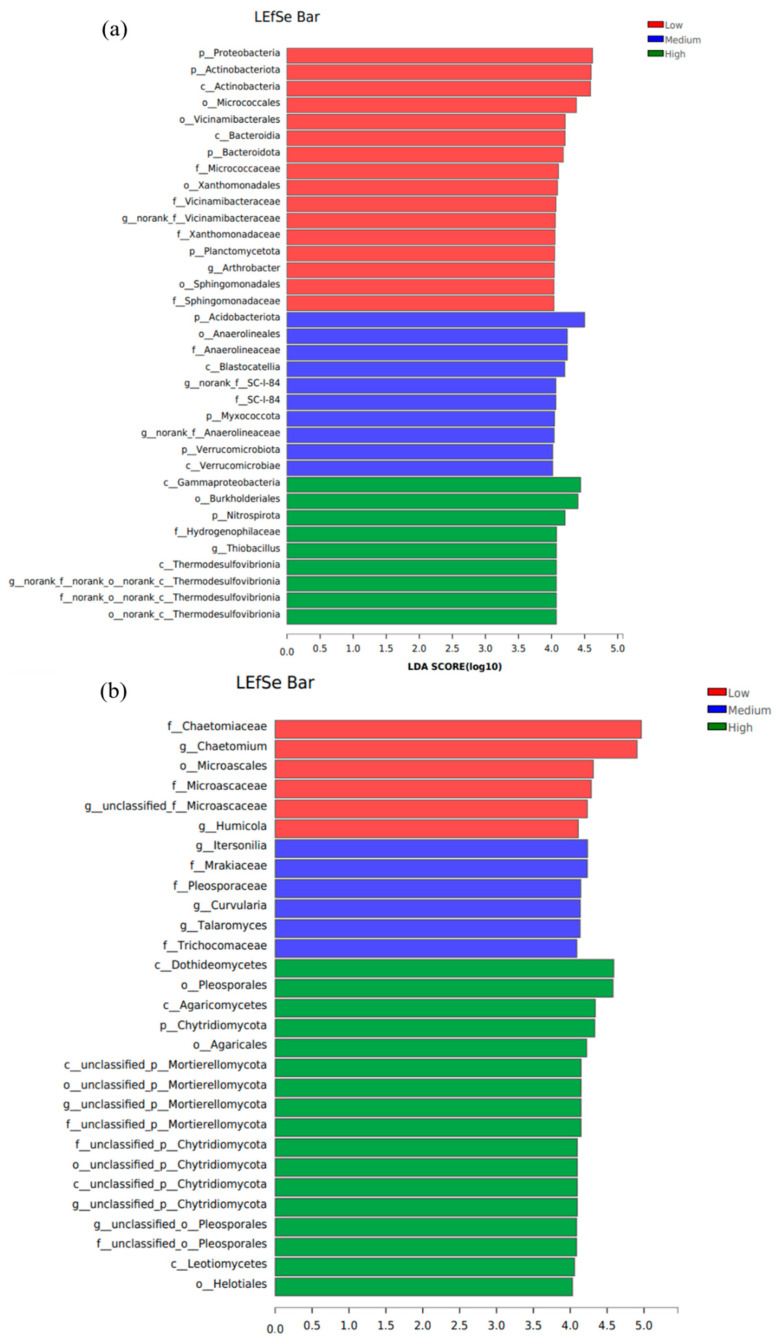
Differential genera of bacteria (**a**) and fungi (**b**) in soils at the low, medium, and high pollution levels.

The Linear Discriminant Analysis Effect Size (LEfSe) method was employed to identify bacterial taxa exhibiting significant differences in relative abundance across different HM pollution levels, with results presented in Figure 7a. When the Linear Discriminant Analysis (LDA) threshold was set to 4.0, a total of six bacterial genera showed statistically significant differences.

As shown in Figure 7a, two dominant bacterial genera were identified in soils under low, medium, and high pollution levels, respectively. Specifically, the differentially abundant genera in low-pollution soils primarily included *Arthrobacter* (phylum Actinobacteria) and *g_norank_f_Vicinamibacteraceae* (phylum Acidobacteriota; without clear taxonomic assignment). In medium-pollution soils, the dominant differential genera were *g_norank_f_SC-I-84* and *g_norank_f_Anaerolineaceae* (both belonging to the phylum Chloroflexi; without clear taxonomic assignment). Soils under high pollution levels were characterized by the genera *Thiobacillus* (phylum Proteobacteria) and *g_norank_f_norank_o_norank_c_Thermodesulfovibrionia* (phylum Nitrospirae; without clear taxonomic assignment).

The LEfSe method was also applied to identify fungal taxa exhibiting significant differences in relative abundance across different HM pollution levels, with results shown in Figure 7b. With the LDA threshold set to 4.0, a total of nine fungal genera showed statistically significant differences. As indicated in Figure 7b, three differentially abundant fungal genera were identified in soils under low, medium, and high pollution levels, respectively. Specifically, the dominant differential genera in low-pollution soils included *g__unclassified_f__Microascaceae* (without clear taxonomic assignment), *Chaetomium*, and *Humicola*, all belonging to the phylum Ascomycota. In medium-pollution soils, the significant genera consisted of *Curvularia* (Ascomycota), *Itersonilia* (Basidiomycota), and *g__unclassified_p__Mortierellomycota* (phylum Mortierellomycota; without clear taxonomic assignment). Soils under high pollution levels were characterized by *Talaromyces*, *g__unclassified_o__Pleosporales*, and *g__unclassified_o__Sordariales*, all within the phylum Ascomycota and lacking clear taxonomic classification.

### 3.6. Relationship Between Soil Properties and Soil Bacterial and Fungal Community Structures

To explore the relationship between environmental factors and microbial communities and to identify the most influential factors, canonical correspondence analysis (CCA) was performed on microbial data and relevant environmental variables. In the resulting CCA biplot, points with different colors and shapes represent sample groups under different HM pollution levels, while red arrows originating from the origin represent different environmental factors. Prior to the correlation analysis between microorganisms and environmental factors, variables were screened using the variance inflation factor (VIF) to eliminate those with strong collinearity.

**Figure 8:**
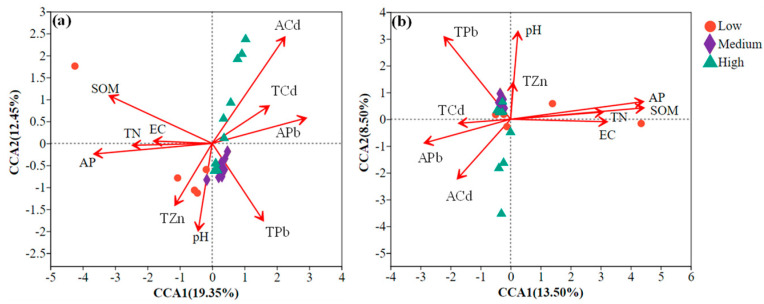
CCA of the relationship between environmental factors and bacterial (**a**) and fungal (**b**) community structures.

The CCA results for the relationship between bacterial communities and environmental factors are presented in Table 5. Available phosphorus, total nitrogen, organic matter, total Pb, available Cd, and available Pb were found to significantly influence bacterial community structure (*p* < 0.05). As shown in Figure 8a, the CCA1 and CCA2 axes together explained 31.8% of the total variation in the bacterial community.

Among these environmental factors, available phosphorus, organic matter, total nitrogen, and electrical conductivity showed positive correlations with each other; total Pb, total Zn, and pH were also mutually positively correlated. Similarly, total Cd, available Cd, and available Pb formed a positively correlated group.

pH was positively correlated with some samples from low and high pollution levels and all samples from the medium pollution level, while it was negatively correlated with the remaining samples. All samples from the low pollution level and some samples from medium and high pollution levels were positively correlated with available phosphorus, organic matter, total nitrogen, and electrical conductivity. These physicochemical properties had a more pronounced impact on samples from low pollution levels compared to those from medium and high pollution levels. Some samples from highly polluted soils showed positive correlations with the concentrations of available Cd, available Pb, and total Cd.

As shown in Table 6, pH, total nitrogen, available phosphorus, organic matter, total Pb, available Cd, and available Pb significantly influenced fungal communities (*p* < 0.05). According to Figure 8b, the CCA1 and CCA2 axes together explained 22.0% of the variation in the fungal community. Among these environmental factors, available phosphorus, organic matter, total nitrogen, and electrical conductivity were positively correlated with each other; total Pb, total Zn, and pH were also positively correlated. Similarly, total Cd, available Cd, and available Pb formed a positively correlated group.

pH was negatively correlated with changes in fungal community composition in some samples from low and high pollution levels, while it was positively correlated with all remaining samples. Meanwhile, community variation in these samples was positively correlated with soil concentrations of available Cd, available Pb, and total Cd. As also observed in Figure 8b, all other fungal samples from medium and high pollution levels exhibited positive correlations with concentrations of Pb, Zn, and Cd. The two samples from the low pollution level showed positive correlations with available phosphorus, organic matter, total nitrogen, and electrical conductivity.

**Figure 9:**
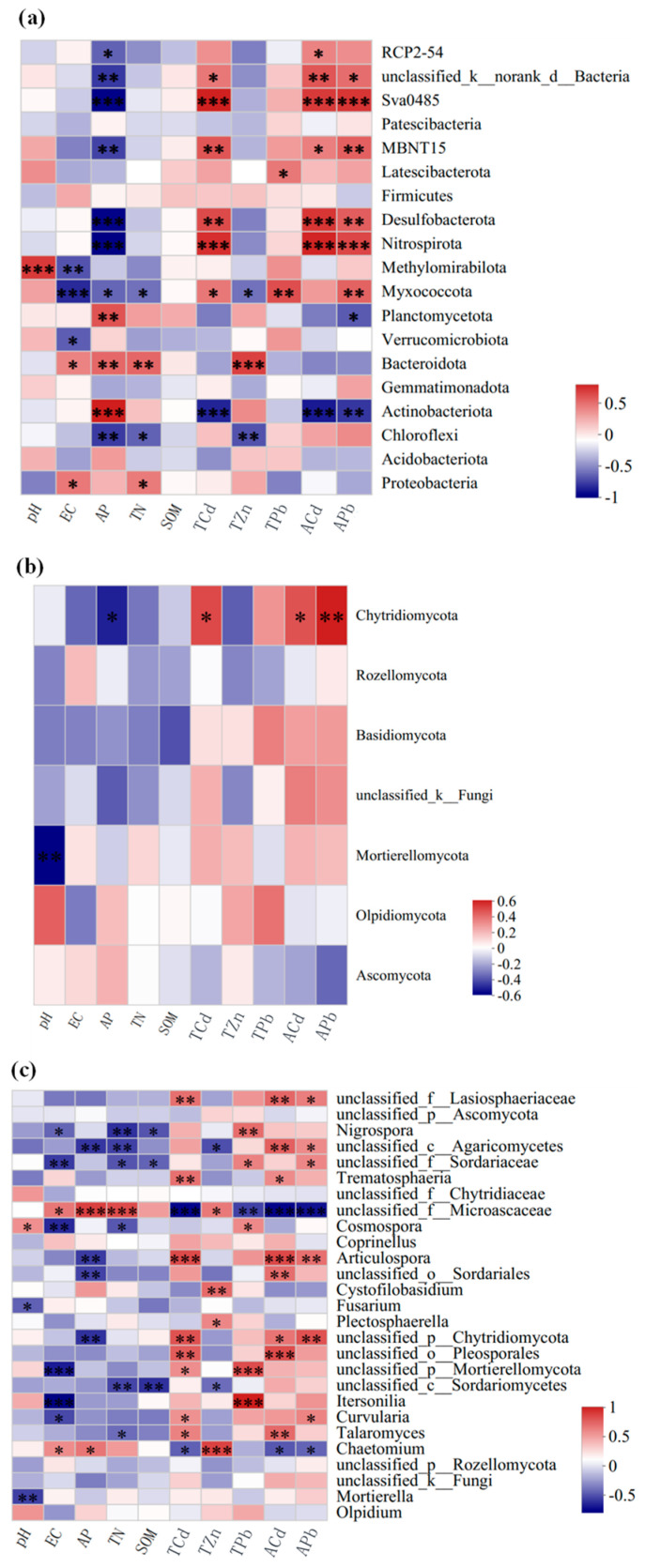
Heatmap of the correlation between environmental factors and bacterial (**a**) and fungal (**b**,**c**) community structures. Note: The significance of the effect is indicated by * *p* < 0.05, ** *p* < 0.01, and *** *p*< 0.001.

A heatmap illustrating the correlation between environmental factors and the top 19 bacterial phyla ranked by total abundance is presented in Figure 9a. Spearman’s rank correlation coefficients were calculated to quantify the associations between environmental factors and bacterial taxa. The magnitude and direction of correlations are represented by color gradients in the heatmap. *Nitrospirota, Desulfobacterota*, *MBNT15*, *Sva0485*, and *unclassified_k__norank_d__Bacteria* had a significantly positive correlation with the concentration of available Cd, available Pb, and total Cd, but a significantly negative correlation with available phosphorus (*p* < 0.05). In contrast, *Actinobacteriota* exhibited an opposite correlation pattern with these factors. *Myxococcota* showed a significantly positive correlation with the concentration of total Pb and available Pb, but a significantly negative correlation with total nitrogen, available phosphorus, and electrical conductivity (*p* < 0.05). *Proteobacteria* demonstrated a significant positive correlation with total nitrogen and electrical conductivity. *Chloroflexi* exhibited a significantly negative correlation with Zn concentration, total nitrogen, and available phosphorus. *Bacteroidota* displayed a significantly positive correlation with Zn concentration, total nitrogen, available phosphorus, and electrical conductivity. *Methylomirabilota* was significantly positively correlated with pH but significantly negatively correlated with electrical conductivity. *Latescibacteria* exhibited a significantly positive correlation with Pb concentration. *Planctomycetes* showed a significantly positive correlation with available phosphorus.

Heatmaps depicting the correlations between environmental factors and the top 7 fungal phyla (Figure 9b) and the top 27 fungal genera (Figure 9c), ranked by total abundance, were generated using Spearman’s rank correlation coefficients. The strength and direction of correlations are represented by color gradients in the heatmaps. At the phylum level, *Chytridiomycota* had a significantly negative correlation with available phosphorus (*p* < 0.05) but a significantly positive correlation with total Cd, available Cd, and available Pb. In contrast, *Mortierellomycota* showed a significantly negative correlation with soil pH (*p* < 0.01). At the genus level, *Olpidium* and *Fusarium* had a significantly negative correlation with soil pH, while *Cosmospora* had a significantly positive correlation with soil pH. *Cosmospora* and *Nigrospora* exhibited a significantly negative correlation with total nitrogen and electrical conductivity, but a significantly positive correlation with total Pb. *Itersonilia* was also significantly positively correlated with total Pb. *Talaromyces*, *Trematosphaeria*, and *Articulospora* exhibited significant positive correlations with total Cd and available Cd. *Articulospora* was significantly positively correlated with available Pb but significantly negatively correlated with available phosphorus. In contrast, *Chaetomium* displayed correlation patterns opposite to those of *Articulospora* and was also significantly positively correlated with total Zn and electrical conductivity. *Plectosphaerella* and *Cystofilobasidium* showed significant positive correlations with total Zn. *Curvularia* exhibited significant positive correlations with total Pb and available Cd, but a significant negative correlation with electrical conductivity.

### 3.7. Core Operational Taxonomic Units in Bacterial and Fungal Communities from Soils with Heavy Metal Pollution

**Figure 10:**
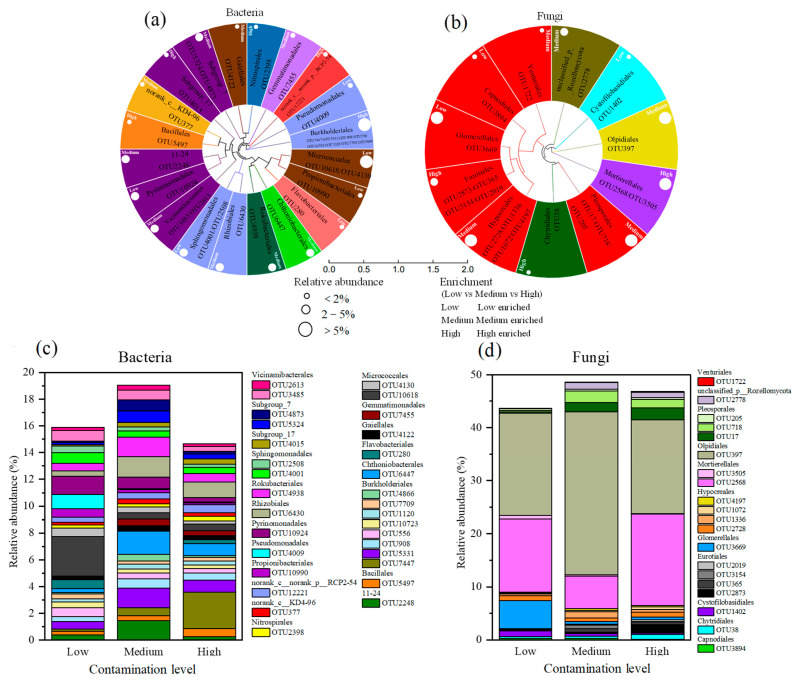
Maximum-likelihood phylogenetic tree (**a**,**b**) and relative abundance (**c**,**d**) of core bacterial and fungal OTUs in soils at the low, medium, and high pollution levels. The bacterial and fungal phyla in (**a**,**b**) are represented by different colors. White circles represent the relative abundance of different samples. Low, Medium, and High represent the enrichment of relative abundance in soil samples at the low, medium, and high pollution levels, respectively. The relative abundance of core OTUs in soil samples at the low, medium, and high pollution levels is shown in (**c**,**d**).

The maximum-likelihood phylogenetic tree of 31 core bacterial operational taxonomic units (OTUs), each with a relative abundance greater than 0.25% and an occurrence frequency of 100% across all treatments, is shown in Figure 10a. These core OTUs were predominantly classified within the phyla *Acidobacteriota*, *Proteobacteria*, and *Actinobacteriota*, and were mostly enriched in samples from the medium pollution level. The relative abundances of these 31 core bacterial OTUs are presented in Figure 10c. The OTUs were primarily assigned to the following taxonomic orders: within *Proteobacteria*—*Burkholderiales*, *Rhizobiales*, *Sphingomonadales*, and *Pseudomonadales*; within *Gemmatimonadota*—*Gemmatimonadales*; within *Acidobacteriota*—*Pyrinomonadales*, *Vicinamibacterales*, *Subgroup_7*, and *Subgroup_17*; within *Actinobacteriota*—*Micrococcales*, *Gaiellales*, and *Propionibacteriales*; within *Bacteroidota*—*Flavobacteriales*; within *Methylomirabilota*—*Rokubacteriales*; within *Nitrospirota*—*Nitrospirales*; within *Firmicutes*—*Bacillales*; and within *Verrucomicrobia*—*Chthoniobacterales*. In low-pollution soils, OTU10618 (*Micrococcales*), OTU10924 (*Pyrinomonadales*), and OTU4009 (*Pseudomonadales*) exhibited the highest relative abundances, at 2.95%, 1.37%, and 1.07%, respectively, while OTU7455 (*Gemmatimonadales*) showed the lowest abundance at 0.08%. Under medium pollution levels, OTU6447 (*Chthoniobacterales*) and OTU6430 (*Rhizobiales*) had the highest relative abundances, reaching 1.74% and 1.54%, respectively, whereas OTU280 (*Flavobacteriales*) had the lowest at 0.05%. In highly polluted soils, OTU7447 (*Burkholderiales*), OTU6430 (*Rhizobiales*), and OTU6447 (*Chthoniobacterales*) displayed the highest abundances, at 2.73%, 1.19%, and 0.88%, respectively, while OTU4009 (*Pseudomonadales*) showed the lowest at 0.10%.

The maximum-likelihood phylogenetic tree of 20 core fungal OTUs, each with a relative abundance greater than 0.1% and an occurrence frequency exceeding 85% across all treatments, is shown in Figure 10b. These core OTUs were predominantly classified within the phylum *Ascomycota* and were mostly enriched in samples from the medium pollution level. The relative abundances of these 20 core fungal OTUs are presented in Figure 10d. The OTUs were primarily assigned to the following taxonomic orders: within *Chytridiomycota*—*Chytridiales*; within *Ascomycota*—*Pleosporales*, *Glomerellales*, *Hypocreales*, *Eurotiales*, *Capnodiales*, and *Venturiales*; within *Olpidiomycota*—*Olpidiales*; within *Mortierellomycota*—*Mortierellales*; and within *Basidiomycota*—*Cystofilobasidiales*. In low-pollution soils, OTU397 (*Olpidiales*), OTU2568 (*Mortierellales*), and OTU3669 (*Glomerellales*) exhibited the highest relative abundances, reaching 19.4%, 13.7%, and 36.7%, respectively. Under medium pollution levels, OTU397 (*Olpidiales*), OTU2568 (*Mortierellales*), and OTU718 (*Pleosporales*) showed the highest abundances, at 30.8%, 6.15%, and 2.20%, respectively. In highly polluted soils, OTU397 (*Olpidiales*), OTU2568 (*Mortierellales*), and OTU17 (*Pleosporales*) displayed the highest relative abundances, at 17.7%, 17.2%, and 2.33%, respectively.

## 4. Discussion 

### 4.1. Relationship Between Heavy Metal Pollution Levels and Basic Physicochemical Properties of Soil

Soil HM pollution levels were found to significantly influence soil physicochemical properties. As HM pollution became aggravated, electrical conductivity, total phosphorus, available phosphorus, available potassium, and total nitrogen all decreased significantly, while available Cd, available Zn, and available Pb increased significantly. These findings align with reports that HM levels in soil could be reduced by applying phosphorus-containing passivators [21]. An increase in soil phosphorus content may lead to the generation of more phosphate radicals in soil colloids, which can precipitate with HM ions, thereby reducing their availability. Appel et al. also found a negative correlation between potassium content and HMs in soil. This may be attributed to competitive adsorption between potassium ions and HM ions such as Cd, resulting in decreased HM retention as potassium levels rise [22]. Kapoor et al. also reported that the penetration of HMs into cell membranes could impair enzyme activity, thereby inhibiting nitrification reaction in soil, which may be responsible for the decreased concentration of total nitrogen with an increase in the concentration of HMs [23]. Additionally, HMs alter the local soil ecosystem and indirectly lead to a reduction in soil nutrients.

This study revealed a positive correlation between soil pH and the concentrations of Zn and Pb (Figure 8a,b), which is consistent with previous research. It has been reported that at low pH, HMs often combine with organic matter to form stable solids, facilitating their immobilization in soil [24]. We also observed a negative correlation between soil organic matter and HM concentrations (Figure 8a,b), supporting demonstrations that increasing organic matter through organic amendments can reduce HM availability in mining soils [25]. The content of soil organic carbon may affect the amount of soil aggregates, which can directly absorb HMs [26]. However, organic matter exhibits different adsorption and solubility patterns for different HMs [27]. This study confirms the interrelationship among soil HM content, chemical properties, and ecosystem processes, which aligns with our initial hypothesis and is supported by previous research [28]. Furthermore, we demonstrated that bacteria and fungi exhibit distinct tolerance thresholds to different HM pollution levels, reflecting their differential capacities for HM absorption and decomposition. Bacteria showed higher sensitivity to HM pollution compared to fungi, a finding consistent with earlier studies [29,30]. Therefore, the selection of microorganisms for bioremediation should be tailored to specific HM pollution levels to enable effective biodegradation.

### 4.2. Relationship Between Heavy Metal Pollution Levels and Bacterial Communities

Areas subjected to HM pollution tend to develop similar dominant microbial species. In this study, *Proteobacteria* were identified as the most dominant phylum in the bacterial community at the sampling site (Figure 4a), which aligns with the predominant microbial taxa reported by Shen et al. (2018) near an industrial area in Zhuzhou, Hunan Province, China [31]. Similarly, Zhao et al. (2019) observed *Proteobacteria* to be the most abundant bacterial phylum in long-term HM-contaminated soils [32]. This prevalence may be attributed to the broad metabolic versatility and degradation capabilities of *Proteobacteria*, as well as their high adaptability to diverse soil environments [33]. *Proteobacteria* are also widely employed in the bioremediation of multi-HM contaminated soils [34]. Other major phyla identified in this study, including *Acidobacteriota*, *Chloroflexi*, *Bacteroidota*, *Planctomycetes*, and *Verrucomicrobia*, have also been reported to exhibit relatively high abundances in HM-polluted soils in previous research [35,36]. Gao et al. (2021b) noted significantly elevated relative abundances of *Actinobacteriota*, *Cyanobacteria*, and *Gemmatimonadota* in HM-contaminated environments [37]. These microbial groups may possess distinct HM resistance mechanisms. For instance, the species in the Actinobacteriota genus, such as *Gaiella* and *Arthrobacter,* typically produce extracellular enzymes and multiple secondary metabolites. These secondary metabolites and enzymes can interact, thereby removing pesticides, HMs, and other foreign compounds [38]. *Bacteroidota* can synthesize all necessary organic compounds using CO_2_. As a category of chemoautotrophic bacteria, they can easily survive in HM-contaminated environments [39,40]. Certain *Acidobacteriota* species can convert complex organic carbon into short-chain fatty acids, which may promote the dissolution or absorption of HMs by hyperenriched plants [41,42]. Consequently, *Acidobacteriota* are generally regarded as highly tolerant to HMs. Long-term exposure to contamination may have selected for HM resistance genes in these bacterial lineages, rendering them less sensitive to metal toxicity [12,43]. The core bacterial OTUs with the highest relative abundances in this study were primarily affiliated with *Acidobacteriota*, *Verrucomicrobia*, *Actinobacteriota*, and *Proteobacteria* (Figure 10a), underscoring the potential of these phyla in the bioremediation of HM-polluted soils.

Different soil bacteria exhibit varying responses to HM pollution, which aligns with the findings of previous studies [37]. As shown in Figure 4a, *Proteobacteria*, *Acidobacteriota*, and *Chloroflexi* maintained relatively high abundances even under high HM pollution levels. In this study, the abundance of *Chloroflexi* increased significantly with increasing HM pollution, consistent with earlier reports. *Chloroflexi* can utilize diverse trophic pathways, including photosynthesis, heterotrophy, photoautotrophy, and chemoautotrophy [44], enabling them to thrive in HM-contaminated soils. These taxa demonstrated the most vigorous growth capacity within the bacterial communities in Pb-Zn contaminated soil. In contrast, the abundances of *Actinobacteriota*, *Bacteroidota*, and *Planctomycetes* decreased as HM pollution intensified. Dominant bacterial phyla in HM-contaminated paddy soils—such as *Verrucomicrobia* and *Planctomycetes*—were significantly negatively correlated with HM concentrations, particularly at medium pollution levels [45]. Consequently, when microbial communities in contaminated soils are exposed to HMs, the diversity and abundance of sensitive species may decline to varying degrees. In contrast, resistant microorganisms adapt to the altered environment, leading to a gradual increase in their abundance [46].

### 4.3. Relationship Between Environmental Factors and Bacterial Communities

Soil physicochemical properties significantly influence bacterial communities. In this study, the bacterial community structure underwent more pronounced changes in highly polluted soils compared to medium- and low-pollution areas (Figure 3a), a finding consistent with CCA (Figure 8a). Both CCA and Pearson correlation heatmap analyses indicated that HMs and soil chemical indicators (e.g., pH, electrical conductivity, organic matter, total nitrogen, and available phosphorus) were closely associated with the relative abundance and diversity of bacterial communities Figure 8a and Figure 9a). Numerous studies have identified pH as a key factor determining bacterial community composition in HM-contaminated soils [47,48]. Certain bacterial genera, such as *Fusarium*, *Blastomonas*, and *Chloroflexus*, exhibit a positive correlation with pH [49]. Other environmental factors, including organic matter, total nitrogen, total phosphorus, and available potassium, have also been reported as major influencers of microbial community structure [45].

Analysis of microbial communities in long-term HM-contaminated soils revealed a significant positive correlation between *Proteobacteria* and Zn and Cu, and a significant negative correlation with organic matter, moisture content, and available potassium [32]. In contrast, this study identified total nitrogen and electrical conductivity as the primary factors associated with *Proteobacteria*. While high concentrations of Cu, Zn, and Pb may not substantially alter bacterial community structure—with nutrient content potentially playing a more critical role [50]—the present results demonstrate that HM concentrations also exert considerable influence on bacterial communities (Figure 8a). These findings highlight the complexity and diversity of bacterial communities across HM pollution gradients and habitat types, underscoring the need for further research integrating multiple environmental variables.

### 4.4. Effects of Environmental Factors and Heavy Metal Pollution Levels on Fungal Community Structure

Soil physicochemical properties and HM pollution levels have significant effects on the structure and composition of fungal communities in soil. In this study, it was found that the relative abundance of *Chytridiomycota* and *Talaromyces* increased significantly with rising HM pollution levels, whereas that of *Chaetomium*, *Plectosphaerella*, *Cystofilobasidium*, *Humicola*, and *Aspergillus* decreased significantly with rising HM pollution levels.

*Chytridiomycota*, *Chaetomium*, and *Talaromyces* were significantly influenced by both HM pollution levels and soil physicochemical properties (*p* < 0.05). The CCA and environmental factor correlation heatmap results further confirmed that HMs and environmental factors are key determinants of fungal community composition (Figure 8b and Figure 9b,c). These results align with previous studies indicating that shifts in microbial community structure can be attributed to multiple factors, including soil properties and toxic pollutants such as HMs [51,52]. Spearman’s rank correlation analysis indicated that nearly all fungal phyla and genera did not exhibit significant negative correlations with soil organic matter (Figure 9b,c), suggesting that fungi may require increased nutrient uptake for metabolism and survival under HM stress [53]. Soil physicochemical properties exerted different effects on fungal communities across different HM pollution levels. This is because different fungi would be screened under different HM pollution levels. In addition, fungi that can survive under different HM pollution levels exhibit differential responses to soil properties. Some soil physicochemical properties can regulate the toxicity of HMs. For example, nutrient elements such as available phosphorus, available potassium, and soil organic matter can facilitate the growth and reproduction of HM-tolerant microorganisms in soil, so that the toxicity caused by HMs can be effectively reduced [50].

In this study, PCoA and community composition analyses (Figure 3b and Figure 4b,c) confirmed variations in fungal community structure across soils with different HM pollution levels, consistent with previous findings [54]. Fungi adapt to varying HM pollution levels by modulating community abundance and structure [32]. The lowest fungal diversity was observed in low-pollution soils, potentially because mild contamination eliminated some metal-sensitive fungi without yet promoting the growth of tolerant taxa [53]. Under increasing pollution, the diversity and abundance of metal-sensitive fungi declined, while metal-tolerant fungi increased after adapting to the altered environment [55]. *Ascomycota*, the most dominant phylum in this study, is widely abundant in soil, which aligns with other reports [56]. Its prevalence may be attributed to metabolic versatility and adaptability to diverse habitats. *Ascomycota* also demonstrates strong HM tolerance, often increasing markedly under moderate pollution [57]. *Mortierella* was identified as the second most abundant genus, followed by *Fusarium* and *Aspergillus* (Figure 4b,c), all considered HM-tolerant fungi [58]. *Fusarium* exhibits a strong capacity for HM accumulation and removal, enabling adaptation to stressful conditions [30]. *Mortierella* contributes to phosphate solubilization and supports arbuscular mycorrhizal fungal colonization [59]. *Aspergillus* shows high tolerance and biosorption capacity for Pb and Cd [60]. The most abundant core fungal OTUs belonged to *Ascomycota* and *Mortierella* (Figure 10b), further supporting their potential for remediating HM-contaminated soils.

## 5. Conclusions

In farmland soils within tailings-contaminated zones, HM pollution levels significantly influenced the key soil physicochemical properties and the β-diversity of both soil bacteria and fungi. With increasing HM pollution levels, the abundances of *Proteobacteria*, *Acidobacteriota*, *Actinobacteriota*, *Bacteroidota*, and *Planctomycetes* decreased, while those of *Chloroflexi*, *Myxococcota*, *Nitrospirota*, and *Desulfobacterota* increased significantly. Similarly, under elevated HM pollution, the relative abundance of *Chytridiomycota* and *Talaromyces* increased significantly, whereas *Chaetomium*, *Plectosphaerella*, *Cystofilobasidium*, *Humicola*, and *Aspergillus* decreased significantly. Core bacterial OTUs were primarily classified within *Proteobacteria*, *Gemmatimonadota*, *Acidobacteriota*, *Actinobacteriota*, *Bacteroidota*, *Methylomirabilota*, *Nitrospirota*, *Firmicutes*, and *Verrucomicrobia*, while core fungal OTUs were mainly affiliated with *Chytridiomycota*, *Ascomycota*, *Olpidiomycota*, *Mortierellomycota*, and *Basidiomycota*. These core microorganisms exhibited strong adaptive capacity to soils across different pollution levels and thus held potential for HM remediation in various contaminated soils. Bacterial and fungal communities were predominantly influenced by HM and nutrients in soils. In conclusion, chronic HM contamination caused the elevated abundance of HM-tolerant microorganisms in highly contaminated soils.

Factors like geographical regions, soil types, and soil physicochemical properties significantly affect microorganisms under HM stress. This study covered a relatively limited range of soil types and conditions; subsequent targeted research and evaluations on specific regions and soils are thus needed. It also lacked sufficient understanding of the related metabolism and functional recovery of different microorganisms during long-term HM contamination, requiring further exploration of their mechanisms of action.

## Figures and Tables

**Table 1:** Risk screening values and risk control values for soil pollution in agricultural land.

	Pollutant Categories	5.5 < pH ≤ 6.5	6.5 < pH ≤ 7.5
Risk screening concentration(mg/kg)	Cd	0.3	0.3
	Pb	90	120
	Zn	200	250
Risk control concentration(mg/kg)	Cd	2.0	3.0
	Pb	500	700
	Zn	/	/

**Table 2:** Soil physicochemical properties and heavy metal concentration under different heavy metal pollution levels.

Contamination Level	* Low	Medium	High
pH	6.42 ± 0.06 a	6.59 ± 0.19 a	6.29 ± 0.18 a
EC (μs/cm)	591 ± 270 a	128 ± 12 b	204 ± 24 b
TP (g/kg)	0.96 ± 0.17 a	0.39 ± 0.01 b	0.38 ± 0.02 b
AP (mg/kg)	55.8 ± 13.3 a	18.2 ± 1.4 b	15.8 ± 1.2 b
AK (mg/kg)	322 ± 34 a	89.2 ± 10.0 b	125 ± 24 b
TN (g/kg)	2.99 ± 0.19 a	2.08 ± 0.06 c	2.44 ± 0.09 b
SOC (g/kg)	69.4 ± 9.3 a	54.4 ± 2.0 b	59.7 ± 2.3 ab
TCd (mg/kg)	0.02 ± 0.01 c	3.12 ± 0.22 b	8.58 ± 1.29 a
TZn (mg/kg)	1014 ± 457 a	457 ± 27 b	628 ± 185 ab
TPb (mg/kg)	21.7 ± 12.0 b	122 ± 7 a	98.6 ± 18.0 a
ACd (mg/kg)	0.004 ± 0.001 c	1.48 ± 0.34 b	3.40 ± 0.59 a
AZn (mg/kg)	4.91 ± 1.61 b	8.79 ± 0.55 b	15.9 ± 2.0 a
APb(mg/kg)	8.32 ± 2.01 b	23.5 ± 2.8 a	25.0 ± 2.2 a

Note: Abbreviations: EC, electric conductivity; TP, total phosphorus; AP, available phosphorus; AK, available potassium; TN, total nitrogen; SOC, soil organic carbon; TCd, soil total Cd; TZn, soil total Zn; TPb, soil total Pb; ACd, soil available Cd; AZn, soil available Zn; APb, soil available Pb. * represents heavy metal pollution levels. The results are presented as the mean of 5 replicates. Different letters represent significant differences among different pollution levels according to Duncan’s multiple range test following significant one-way ANOVA (*p* < 0.05). Same letters indicate no significant difference, while different letters indicate a significant difference; specifically, “a” denotes the largest value, “b” an intermediate one, and “c” the smallest.

**Table 3:** Adonis results of bacterial samples under different heavy metal pollution levels.

		Df	*F*	*R^2^*	*p*
Contamination level	Low–High	1	3.93	0.247	0.003
	Low–Medium	1	5.10	0.317	0.001
	Medium–High	1	2.78	0.156	0.003

**Table 4:** Adonis results of fungal samples under different heavy metal pollution levels.

		Df	*F*	*R^2^*	*p*
Contamination level	Low–High	1	1.92	0.138	0.022
	Low–Medium	1	2.38	0.178	0.006
	Medium–High	1	1.75	0.104	0.063

**Table 5:** Permutation test results of CCA for the relationship between bacterial communities and environmental factors.

	CCA1	CCA2	*r^2^*	*p*
pH	−0.2222	−0.9750	0.203	0.125
EC	−0.9996	0.0293	0.163	0.156
AP	−0.9981	−0.0618	0.701	0.001
TN	−0.9999	−0.0157	0.310	0.035
SOM	−0.9508	0.3097	0.586	0.015
TCd	0.9079	0.4192	0.191	0.115
TZn	−0.6510	−0.7591	0.162	0.171
TPb	0.6865	−0.7271	0.281	0.040
ACd	0.6980	0.7161	0.557	0.001
APb	0.9827	0.1852	0.453	0.001

**Table 6:** CCA results of the correlation between fungal communities and environmental factors.

	CCA1	CCA2	*r^2^*	*p*
pH	0.0738	0.9973	0.357	0.021
EC	0.9995	−0.0319	0.356	0.090
AP	0.9903	0.1392	0.698	0.002
TN	0.9967	0.0810	0.328	0.024
SOM	0.9960	0.0889	0.695	0.001
TCd	−0.9967	−0.0815	0.098	0.350
TZn	0.0566	0.9984	0.056	0.398
TPb	−0.5977	0.8017	0.487	0.002
ACd	−0.6357	−0.7719	0.267	0.048
APb	−0.9591	−0.2831	0.308	0.025

## Data Availability

The original contributions presented in this study are included in the article. Further inquiries can be directed to the corresponding author.
